# Evaluation of Aerosol Particle Leak and Standard Surgical Mask Fit With 3 Elastomeric Harness Designs

**DOI:** 10.1001/jamanetworkopen.2021.45811

**Published:** 2022-01-31

**Authors:** Jeannette Ingabire, Hannah McKenney, Charles Sebesta, Krishna Badhiwala, Caleb Kemere, Sahil Kapur, Jacob T. Robinson

**Affiliations:** 1 Department of Electrical and Computer Engineering, Brown School of Engineering, Rice University, Houston, Texas; 2 Systems, Synthetic, and Physical Biology PhD Program, Institute of Biosciences and Bioengineering, Rice University, Houston, Texas; 3 Division of Surgery, The University of Texas MD Anderson Cancer Center, Houston; 4 Department of Plastic Surgery, The University of Texas MD Anderson Cancer Center, Houston

## Abstract

This comparative effectiveness study evaluates aerosol particle leak and standard surgical mask fit with 3 elastomeric harness designs tested on mannequin heads and human participants using US Occupational Safety and Health Administration N95 fit factor requirements.

## Introduction

Because COVID-19 can be transmitted by airborne aerosols, the use of masks that both effectively filter particles and fit snugly to the face is particularly important.^1^ It is recommended that doctors and other health care professionals use N95 respirators instead of surgical masks while performing high-risk, aerosol-generating procedures.^1,2^ However, early in the COVID-19 pandemic in 2020, N95 respirators were difficult to obtain because of a global shortage of personal protective equipment.^3^ Standard surgical masks tend to have a poor fit, resulting in their limited ability to protect the wearer against viral aerosolized droplets.^3,4^ The US Occupational Safety and Health Administration (OSHA) uses an ambient aerosol condensation nuclei counter quantitative fit testing protocol to evaluate N95 respirator fit.^5^ Here, we developed 3 elastomeric harness designs that allow users to meet the OSHA N95 fit factor requirement (FFR) when worn with a standard surgical mask.

## Methods

This comparative effectiveness study was approved by the institutional review boards of Rice University and The University of Texas MD Anderson Cancer Center. Written informed consent was obtained from all study participants. The study followed the International Society for Pharmacoeconomics and Outcomes Research (ISPOR) reporting guideline.

For this study, small and large mannequin heads corresponding to National Institute for Occupational Safety and Health anthropometric data on respirator users were printed 3-dimensionally.^6^ We designed 3 harnesses with Illustrator software (Adobe), with an emphasis on improving the seal around the nasal sidewalls, around the cheeks, and under the chin (eFigure 1 in the Supplement). Harness effectiveness was tested with the mannequin heads; a platform was designed to model human inhalation and exhalation processes, which were captured using infrared camera recordings (eFigure 2 in the Supplement).

Quantitative fit tests were conducted with human participants at a research institution (Rice University) and a cancer center (The University of Texas MD Anderson Cancer Center). Through flyers sent to the Rice University Graduate School of Engineering network and to health care professionals at The University of Texas MD Anderson Cancer Center, we recruited participants between the ages of 21 and 65 years who did not present symptoms of or have a positive COVID-19 test result. The 8026 Particle Generator (TSI Inc) was used to supplement the ambient particle count in the room. The quantitative fit tests were recorded using the 830 PortaCount Pro Respirator Fit Tester (TSI Inc).

Health care workers were given a survey to complete after using the harness to provide feedback on the harness and user comfort. The fit factor data from all participants wearing different harness designs were computed and plotted in MATLAB (MathWorks). Mean fit factors were also calculated in MATLAB.

## Results

Among 18 participants from Rice University, 11 were women and 7 were men. Among 21 participants from The University of Texas MD Anderson Cancer Center, 11 were women and 10 were men. Data on participant age were only collected at 1 site and are therefore not reported.

**Figure 1.**
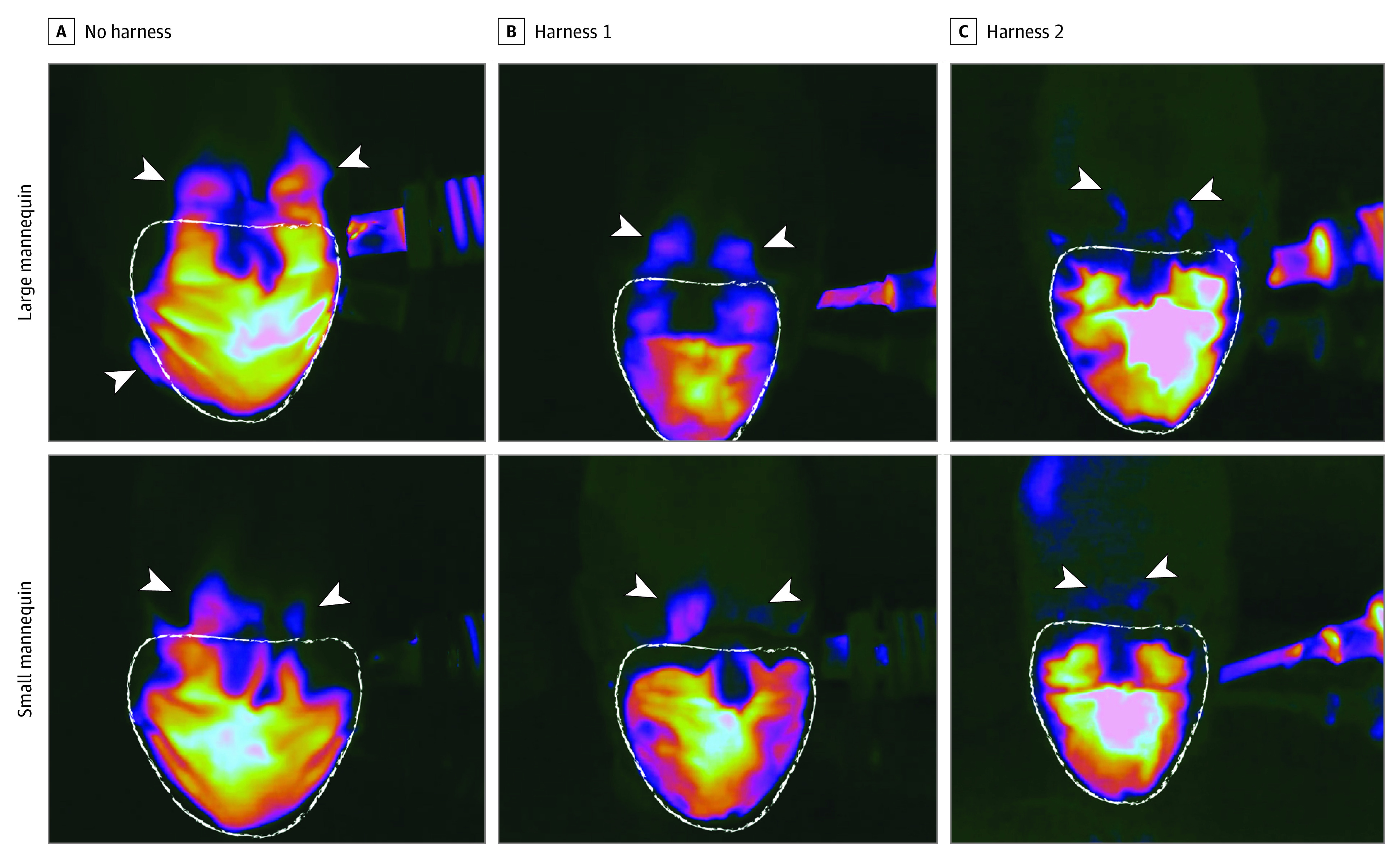
Infrared Imaging of Exhalation Simulations in Small and Large Mannequin Heads A-C, Mannequin head wearing a standard surgical mask only (A), a surgical mask and harness design 1 (B), and a surgical mask and harness design 2 (C). Arrows show the areas most prone to particle leaking (nasal sidewalls).

Infrared imaging showed hot spots around the eyes and nasal sidewalls when the mannequin heads were wearing only the surgical masks, indicating significant air leakage. This air leakage was reduced when harness design 1 was worn over the standard surgical mask (Figure 1B). Infrared imaging of the mannequin heads wearing design 2, which included additional material along the nasal sidewalls, over the surgical mask showed reduced hot spots around the eyes and nasal sidewalls, indicating a better fit (Figure 1C).

**Figure 2.**
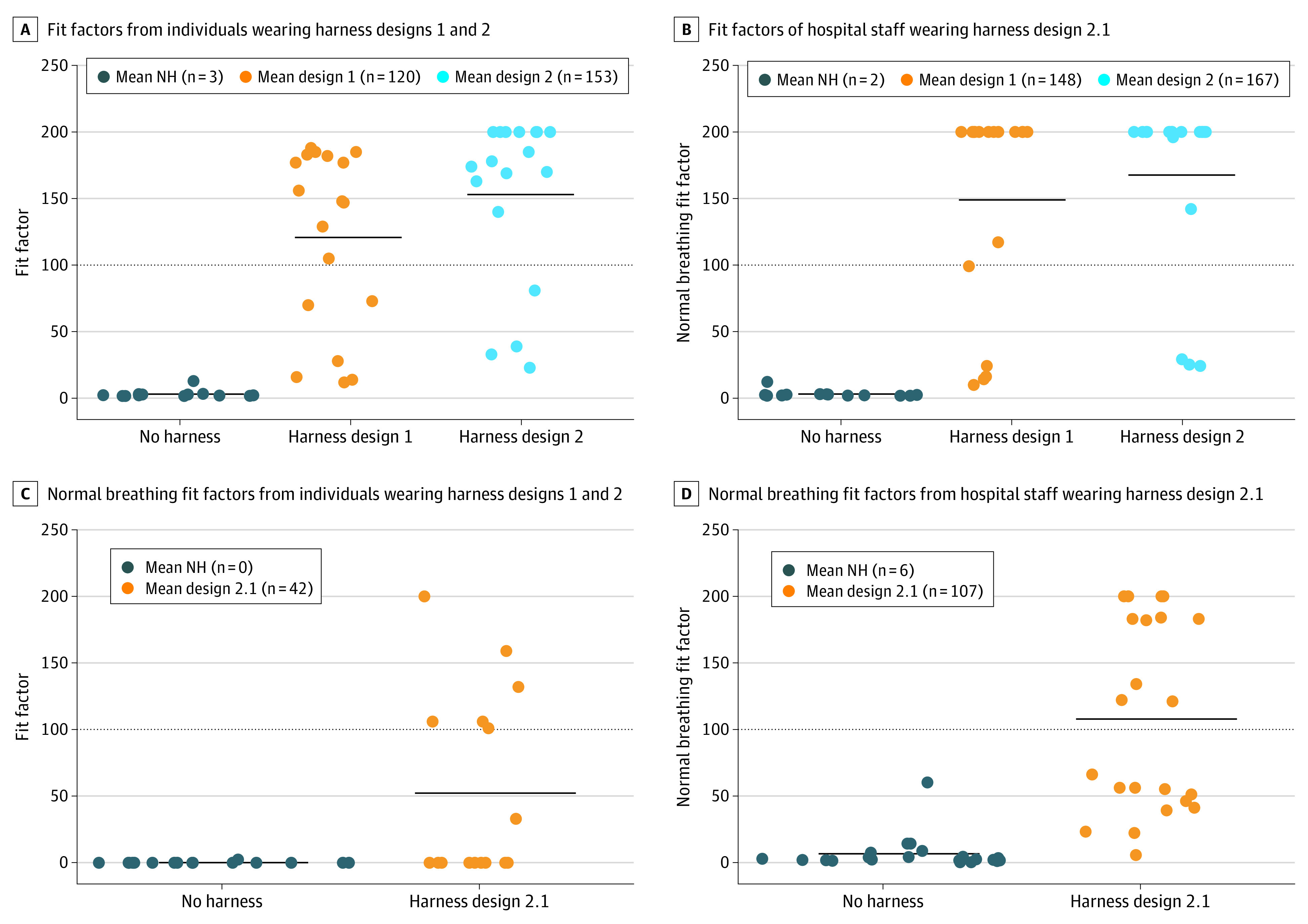
Fit Factors for Elastomeric Harnesses Designs A and B, Overall fit-test scores from participants wearing a standard surgical mask and harness design 1 or 2 (A) and from hospital staff wearing a surgical mask and harness design 2.1 (B). C and D, Normal breathing-test scores of participants wearing a surgical mask and harness designs 1 or 2 (C) and hospital staff wearing a surgical mask and harness design 2.1 (D). NH indicates no harness.

Fit tests for designs 1 and 2 were performed at Rice University and feedback was obtained. As expected, none of the 18 participants wearing the surgical mask alone met the OSHA N95 FFR score of 100 (Figure 2A). When fitted with design 1 over the surgical mask, 12 of the 18 participants (66.7%) achieved a passing FFR score of greater than 100. When fitted with design 2 over the surgical mask, 14 of the 18 participants (77.8%) achieved a passing FFR score (Figure 2A).

Based on the feedback from health care workers, design 2 was modified to reduce the amount of material along the nasal sidewalls to prevent disruption of the user’s field of view while also reducing air leakage (design 2.1). Design 2.1 was tested on 21 perioperative hospital staff members at at The University of Texas MD Anderson Cancer Center. Ten participants (47.6%) passed the fit test while wearing the surgical mask and harness design 2.1 (Figure 2B). The addition of the harness was associated with an almost 15-fold improvement in mean FFR (Figure 2D). The mean (SD) normal breathing fit factor of designs 1, 2, and 2.1 was 148 (78), 167 (66), and 107 (72), respectively.

## Discussion

The results of this comparative effectiveness study suggest that a low-cost, easy-to-manufacture elastomeric harness may improve the fit and protection of a standard surgical mask. Infrared imaging and OSHA-approved fit testing were used to improve the harness design and optimize both fit and user comfort. The use of rubber-band harnesses with surgical masks has been reported, but they lack the consistency and comfort that can be achieved with manufactured harnesses.^5^

A limitation of this study is that we did not evaluate surgical mask fit without a harness for all participants. This study highlights how multidisciplinary short-run manufacturing capability within academic institutions accelerates the application of engineering solutions to health care challenges. Future work should explore other manufacturing techniques such as injection molding, which can make the nasal sidewall seal more comfortable and easier to assemble.

## Notes

Supplement. **eFigure 1.** Improving Standard Surgical Mask Fit by Using an Elastomeric Harness **eFigure 2.** Breathing Simulation Using a Mannequin Head
