# Supplementary material for: Evaluation of Aerosol Particle Leak and Standard Surgical Mask Fit With 3 Elastomeric Harness Designs
Source: JAMA Netw Open. 2022 Jan 31;5(1):e2145811. doi: 10.1001/jamanetworkopen.2021.45811 (PMC8804914; doi:10.1001/jamanetworkopen.2021.45811)
Supplement: Supplement. — eFigure 1. Improving Standard Surgical Mask Fit by Using an Elastomeric Harness eFigure 2. Breathing Simulation Using a Mannequin Head [file jamanetwopen-e2145811-s001.pdf]

Supplementary Online Content

Ingabire J, McKenney H, Sebesta C, et al. Evaluation of aerosol particle leak and standard surgical mask fit with 3 elastomeric harness designs. *JAMA Netw Open*. 2022;5(1):e2145811. doi:10.1001/jamanetworkopen.2021.45811

eFigure 1. Improving Standard Surgical Mask Fit by Using an Elastomeric Harness

eFigure 2. Breathing Simulation Using a Mannequin Head

This supplementary material has been provided by the authors to give readers additional information about their work.

© 2022 Ingabire J et al. *JAMA Network Open*.

eFigure 1. Improving Standard Surgical Mask Fit by Using an Elastomeric Harness

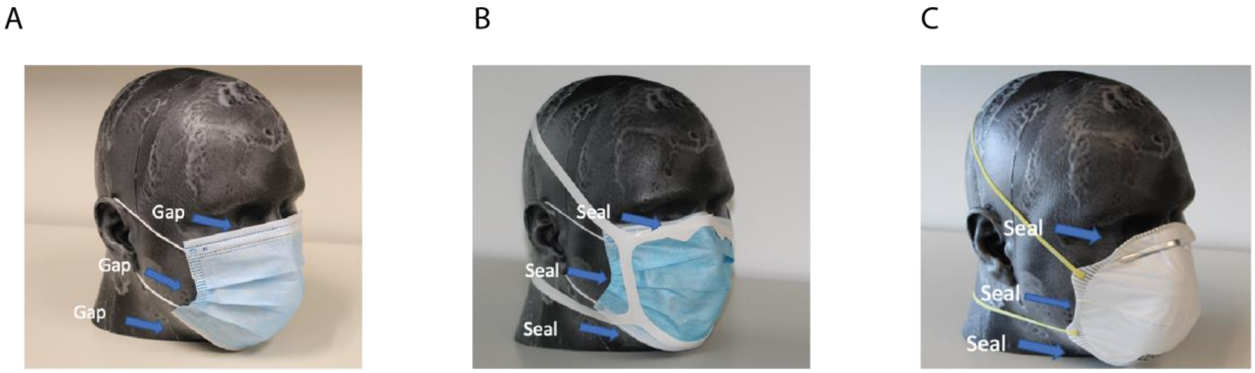

Supplemental eFigure 1: Improving the fit of a surgical mask using elastomeric harness. NIOSH 3D-printed large mannequin head wearing surgical mask alone (A), surgical mask with Harness Design 1 (B), and N-95 respirator. .

© 2022 Ingabire J et al. *JAMA Network Open*.

eFigure 2. Breathing Simulation Using a Mannequin Head

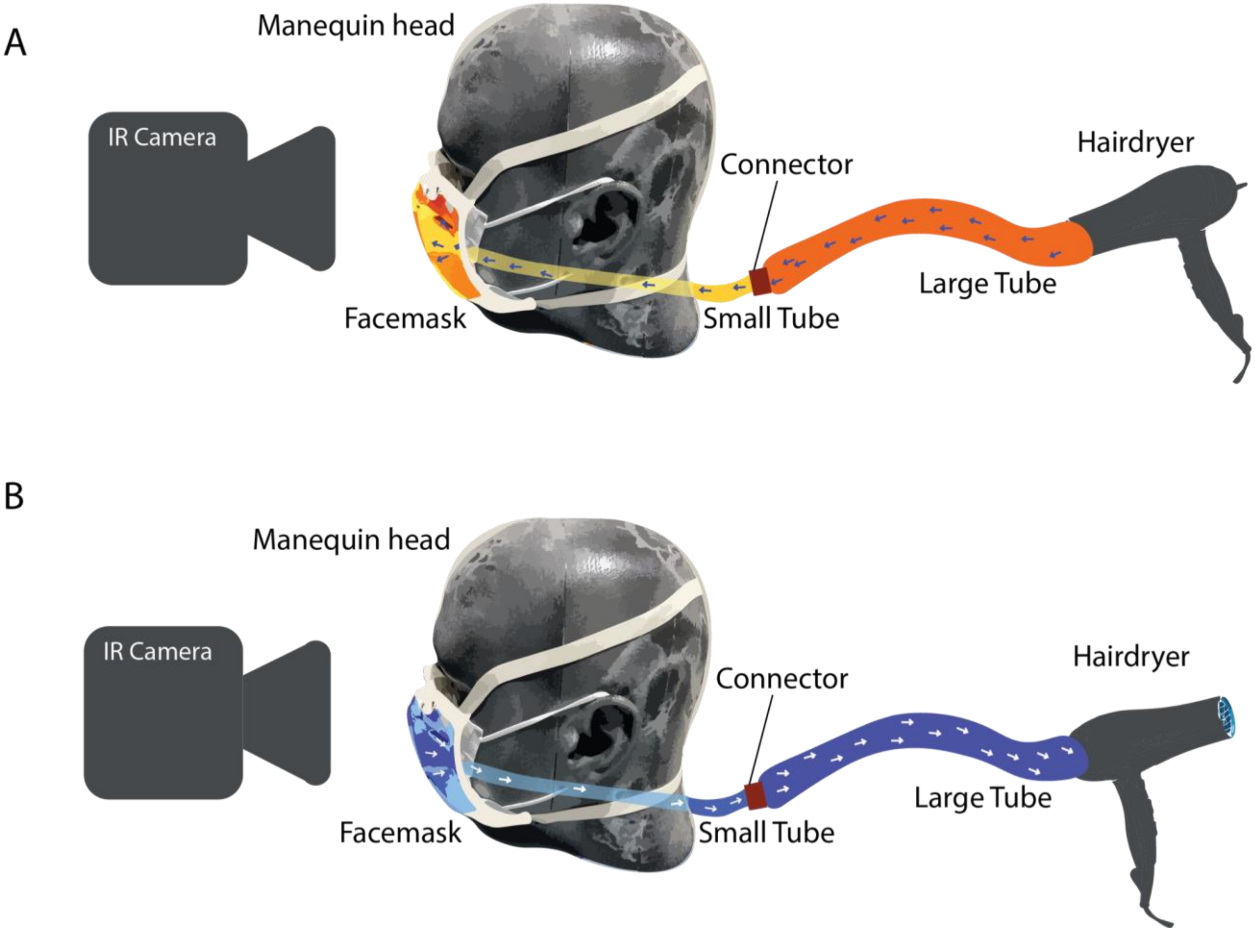

Supplemental eFigure 2: Mannequin Heads breathing simulation of exhalation (**A**) and Inhalation (**B**). Hairdryer introduces warm air into a large tube, which connects to a smaller tube directly in contact with the mannequin mouth. Warm air propagates inside the mask, and the seal efficiency is assessed using an IR camera. The protocol is performed for different mannequin sizes.

© 2022 Ingabire J et al. *JAMA Network Open*.
